# Early blood glucose profile and neurodevelopmental outcome at two years in neonatal hypoxic-ischaemic encephalopathy

**DOI:** 10.1186/1471-2431-11-10

**Published:** 2011-02-04

**Authors:** Montasser Nadeem, Deirdre M Murray, Geraldine B Boylan, Eugene M Dempsey, Cornelius A Ryan

**Affiliations:** 1 Neonatal Intensive Care Unit, Cork University Maternity Hospital, Cork, Ireland; 2 Department of Paediatrics & Child Health, University College Cork, Cork, Ireland

## Abstract

**Background:**

To examine the blood glucose profile and the relationship between blood glucose levels and neurodevelopmental outcome in term infants with hypoxic-ischaemic encephalopathy.

**Methods:**

Blood glucose values within 72 hours of birth were collected from 52 term infants with hypoxic-ischaemic encephalopathy. Hypoglycaemia [< 46.8 mg/dL (2.6 mmol/L)] and hyperglycaemia [> 150 mg/dL (8.3 mmol/L)] were correlated to neurodevelopmental outcome at 24 months of age.

**Results:**

Four fifths of the 468 blood samples were in the normoglycaemic range (392/468:83.8%). Of the remaining 76 samples, 51.3% were in the hypoglycaemic range and (48.7%) were hyperglycaemic. A quarter of the hypoglycaemic samples (28.2%:11/39) and a third of the hyperglycaemic samples (32.4%:12/37) were recorded within the first 30 minutes of life. Mean (SD) blood glucose values did not differ between infants with normal and abnormal outcomes [4.89(2.28) mmol/L and 5.02(2.35) mmol/L, p value = 0.15] respectively. In term infants with hypoxic-ischaemic encephalopathy, early hypoglycaemia (between 0-6 hours of life) was associated with adverse outcome at 24 months of age [OR = 5.8, CI = 1.04-32)]. On multivariate analysis to adjust for grade of HIE this association was not statistically significant. Late hypoglycaemia (6-72 hours of life) was not associated with abnormal outcome [OR = 0.22, CI (0.04-1.14)]. The occurrence of hyperglycaemia was not associated with adverse outcome.

**Conclusion:**

During the first 72 hours of life, blood glucose profile in infants with hypoxic-ischaemic encephalopathy varies widely despite a management protocol. Early hypoglycaemia (0-6 hours of life) was associated with severe HIE, and thereby; adverse outcome.

## Background

Hypoxic-ischaemic encephalopathy (HIE) remains an important cause of neonatal death and long-term neurodisability [1]. Goals of management have been to maintain normoxaemia, normocapnia, normoglycaemia and normal blood pressure to avoid or ameliorate secondary cerebral injuries [2].

Neonatal hypoglycaemia, independent of HIE, has been associated with adverse outcome in both term and preterm infants [3-5]. However, no conclusive evidence on the severity and duration of hypoglycaemia causing brain damage has been reported [6,7]. Basu et al showed that the degree of hypoglycaemia was correlated to the severity of HIE in term asphyxiated newborns [8]. In term infants with severe fetal acidemia, an association between early adverse outcome and hypoglycaemia on the first blood sample was reported by Salhab et al [9]. These studies did not address long-term neurodevelopmental outcome. The mechanism of hypoglycaemic brain injury has been examined in animal models. Hypoglycaemia decreases the cerebrovascular response to hypoxia and increases cerebral superoxide production and aspartate levels into the brain extracellular space resulting in neuronal necrosis [10-12].

Hyperglycaemia is associated with adverse outcome in premature infants, in critically ill children and in adult patients with stroke [13-18]. In extremely low birth weight infants and in patients in paediatric intensive care unit, controlling elevated blood glucose levels by controlled insulin infusion was associated with good short-term outcome [19,20]. However, no data on long-term neurodevelopmental outcome relating to hyperglycaemia in neonatal HIE has been reported [19,20]. The mechanism of hyperglycaemic brain damage is thought to be related to neuronal cell apoptosis following reperfusion with high level of substrate (glucose) in an ATP- deleted cell. In an animal model, hyperglycaemia following hypoxic-ischaemic insult decreases fetal brain ATP and oxygen consumption and increases thickness of vascular endothelium with foci of infarction [21-23].

In the absence of long-term neurodevelopmental outcomes on the effects of early hypoglycaemia and hyperglycaemia in HIE, the aim of this study was to describe the early blood glucose profile and to determine whether hypoglycaemia and hyperglycaemia in the 72 hours of birth were associated with adverse neurodevelopmental outcome at 24 months of age in infants with HIE.

## Methods

This study was a retrospective analysis of a prospective cohort of babies with HIE, none of whom received therapeutic hypothermia. It was conducted in a large maternity hospital with an annual delivery rate of approximately 5000 babies. Ethical approval was obtained from Cork University Hospital Research Ethics Committee. Informed consent was obtained from all parents of the infants who were enrolled in the study. We examined the medical notes of a cohort of 55 term infants with HIE, recruited prospectively at birth between May 2003 and December 2005. Term infants were recruited to the cohort if they fulfilled 2 or more of the following criteria:

• Initial capillary or arterial pH < 7.1

• Apgar score < 5 at 5 minutes

• Initial capillary or arterial lactate > 7 mmol/l

• Abnormal neurology or clinical seizures.

Demographics and data related to neonatal course, ventilation variables and the arterial, capillary and venous blood glucose values of the first 3 days of life were retrieved from the medical notes. All infants with HIE had blood glucose levels checked within the first 30 minutes after delivery. Blood glucose values were collected from either arterial, venous, or capillary samples. An on-site analyser (Radiometer) was used to measure blood glucose levels. Hypoglycaemia and hyperglycaemia were defined as blood glucose levels < 46.8 mg/dL (2.6 mmol/L) and > 150 mg/dL (8.3 mmol) respectively [5,13].

Hypoglycaemia was treated with an initial parenteral bolus of 2 mL/kg of a 10% dextrose solution over one minute. Blood glucose levels were rechecked 30 minutes to 1 hour later according to the severity of hypoglycaemia. Dextrose infusion rate or concentration was adjusted to maintain blood glucose level > 50 mg/dL (2.8 mmol/L). In infants with hyperglycaemia, the rate or concentration of glucose infusion was adjusted to maintain blood glucose within normal levels.

Demographic details and clinical data were recorded in each case. Clinical grade of encephalopathy was assigned using a Sarnat score at 24 hours of age. Neurodevelopmental outcome was assessed at 24 months using the Revised Griffith's scales of Mental Development [24]. Adverse outcome was defined as death, a Griffith's Quotient (GQ) less than 87, or significant motor disability. Statistical analysis was performed using SPSS version 14.0 for Windows. Summary measures were calculated and are reported as mean and standard deviation (SD**) **or median and (range). Spearman correlation was used to explore the differences in categorical variables. Univariate and multivariate logistic regression models were used to estimate odds ratios and 95% confidence intervals. A p value <0.05 was considered statistically significant.

## Results

### Demographics (table 1)

**Table 1:** Demographic data

Total number of infants, n	52
Male, n (%)	34/52 (65.4%)

Infants required ETT at delivery, n (%)	32/52 (61.5%)

Infants required CPR at delivery, n (%)	13/52 (25%)

Birth Weight, median (range)	3.54(1.83-5.04)

Gestational age (weeks+days), median (range)	40+1(35+6-42+1)

Apgar at 1 minute, median (range)	4 (0-9)

Apgar at 5 minutes, median (range)	6 (2-10)

Blood glucose, median (range)	4.5 mmol/L [81 mg/dL], (0.3-17 mmol/L [5.4-306 mg/dL])

Abnormal outcome, n (%)	21/52 (40.4%), of those 2 infants had mild, 10 had moderate and 9 had severe HIE

Seizures	12/52 (23%)

Mortality	Mild HIE: 0/25
	Moderate HIE: 2/18 (11%)
	Severe HIE: 2/9 (22%)

Apgar scores were not available in 4 infants with planned home deliveries; ETT = Endotracheal tube; CRP = Cardiopulmonary resuscitation.

Fifty-two of the 55 infants completed follow up to 24 months. Mean (SD) gestational age was 39 weeks+1 day (1.5 weeks), mean (SD) birth weight was 3.45 (0.58) Kgs. Four infants were delivered at home, and therefore accurate details of Apgar scores and resuscitation details were not available. More than 1 bolus of dextrose infusion was required in four infants. All infants who required increased glucose infusions experienced stable blood glucose within 2 hours of birth.

### 72-hour glucose profile

In total, 468 blood glucose samples were analyzed. Four out of 5 samples were in the normoglycaemic range (392/468: 83.8%). Of the remaining 76 samples, half (51.3%) were in the hypoglycaemic range and less than half were hyperglycaemic (48.7%).

**Figure 1:**
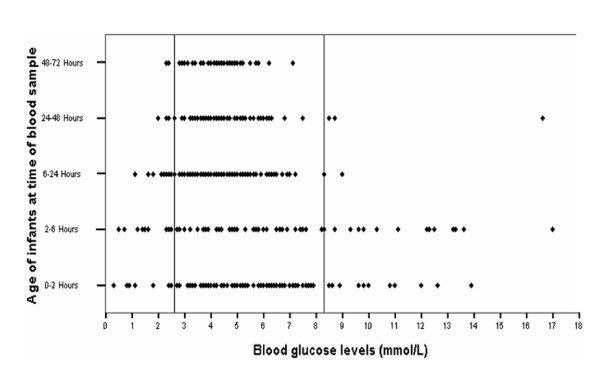
**Distribution of blood glucose levels (mmol/L) according to the timing of blood glucose sample after delivery**. Horizontal lines represent normal range of blood glucose.

The median timing of initial blood glucose sampling was 25 and 80 minutes after birth (range 9-30 and 70-100 minutes) in inborn and outborn infants respectively. More than one third of all blood samples [177/468 (37.8%)] were documented within the first 6 hours of birth. A quarter of the hypoglycaemic (28.2%: 11/39) and a third of the hyperglycaemic samples (32.4%:12/37) were recorded on the first blood samples. Fifty percent (20/39) of the hypoglycaemic and 90% (33/37) of the hyperglycaemic samples were recorded within the first 6 hours of life (figure 1).

Of the 52 infants in the cohort, mild, moderate and severe HIE was seen in 25, 18 and 9 infants, respectively. The mean (SD) number of blood glucose samples, taken in the first 72 hours, was 5.9 (2.9), 11.5 (5.7) and 13.1 (3.6) in infants with mild, moderate and severe HIE, respectively.

### Mean glucose levels and outcome

**Figure 2:**
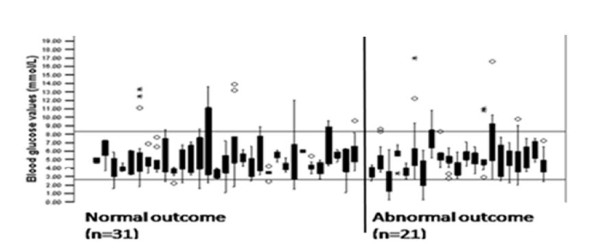
**Distribution of blood glucose levels (mmol/L) in individual infants (n = 52) by neurodevelopmental outcome at 24 months**. Horizontal lines represent normal range of blood glucose. The boxes and whiskers represent the ranges between 25^th ^and 75^th ^percentiles and between 10^th ^and 90^th ^percentiles respectively. The circles (outliers) represent cases with values that are between 1.5 and 3 box lengths from either end of the box. Asterisks (extremes) represent cases with values more than 3 box lengths from rather end of the box.

Abnormal neuro-developmental outcome at 24 months of age was documented in 21 (40.4%) infants. Mean (SD) blood glucose values did not differ between infants with normal or abnormal outcomes [4.89 (2.28) mmol/L and 5.02 (2.35) mmol/L, p value = 0.15] respectively. The distribution of blood glucose levels (mmol/L) in individual infants according to outcome is depicted in figure 2.

### HIE grade, glucose profile and outcome

Mean (SD) of blood glucose level was 4.7 (1.8), 4.9 (2.4) and 5.3 (2.5) mmol/L in infants with mild, moderate and severe HIE respectively, (p value 0.10). The occurrence of early hypoglycaemia correlated significantly with severe HIE (p = 0.012). Twenty-five infants had mild HIE, of whom 23/25 (92%) had a normal outcome. Moderate HIE occurred in 18 infants, of whom 10/18 (55.6%) had abnormal outcome. The nine infants with severe HIE all had an abnormal outcome.

### Infants with normoglycaemia, hypoglycaemia and hyperglycaemia and outcome (table 2)

**Table 2:** Relationship between blood glucose levels and adverse outcome in term infants with HIE (n = 52; however in 7 infants there were both hypoglycaemic and hyperglycaemic episodes documented, explaining the overlap)

	No. of infants (n = 52)	No. of infants With abnormal outcome	Risk of adverse outcome OR (CI)
Normoglycaemia	24/52	9/24 (37.5%)	OR = 0.8, (CI = 0.26-2.44)

Early hypoglycaemia (0-6 hours of life)	8/52	6/8 (75%)	OR = 5.8, (CI = 1.04-32)

Late hypoglycaemia (6-72 hours of life)	12/52	2/12 (8.3%)	OR = 0.22, (CI = 0.04-1.14)

Early hyperglycaemia (0-6 hours of life)	11/52	4/11 (36.4%)	OR = 0.81, (CI = 0.2-3.2)

Late hyperglycaemia (6-72 hours of life)	4/52	2/4 (50%)	OR = 1.53, (CI = 0.2-11.8)

The relationship between early (0-6 hours of life) and late (6-72 hours of life) blood glucose levels and adverse outcome at 24 months of age in infants with HIE is presented in table 2. Early hypoglycaemia (0-6 hours of life) was associated with adverse outcome (OR = 5.8, (CI = 1.04-32). Late hypoglycaemia (between 6-72 hours of life) and early and late hyperglycaemia (0- 6 and 6-72 hours of life, respectively) were not associated with adverse outcome (table 2). Univariate followed by multivariate analysis had been performed.

By univariate analysis, three variables were associated with adverse outcome: early hypoglycaemia, Apgar score ≤ 5 at five minutes and severe HIE (p value 0.025, 0.035 and <0.001 respectively). Moderate HIE, Apgar score at 1 minute, number of blood samples per subject and the requirement for intubation at birth were not significantly associated with adverse outcome. On multivariate analysis only moderate (p = 0.024) and severe (p < 0.001) HIE grade remained significantly associated with adverse outcome.

## Discussion

This study is the first to describe the blood glucose profile during the first 72 hours of life in infants with HIE. Despite a protocolised-driven approach to detecting and treating abnormal blood sugars following perinatal asphyxia in our unit, sustained normoglycaemia in the first 72 hours of birth was observed in only half of our infants with HIE.

What does the glucose profile in HIE tell us? Abnormal blood glucose values were observed in 1 out of 6 samples. Less than a third of hypoglycaemia and hyperglycaemia episodes were observed on the first blood sample taken within a half-hour of birth. However, half of hypoglycaemic samples and 90% of hyperglycaemic samples occurred within 6 hours of birth. These findings suggest that many asphyxiated babies, in addition to facing an hypoxic-ischaemic insult, are concurrently experiencing significant variations in blood sugars in the early newborn period. The hypoglycaemic episodes may be due to perinatal depletion of glycogen stores (many asphyxiated babies are post mature), whilst the hyperglycaemic episodes may reflect the release of stress-related hormones. In addition, since variations in blood glucose levels persist throughout the first 72 hours, this study indicates that regular measurements of the blood glucose should continue throughout this period.

In our study, early hypoglycaemia was associated with an increased risk of adverse outcome at 24 months of age in infants with HIE. However, when corrected for grade of HIE this was no longer significant. Our results are consistent with the findings of Salhab et al. In that study, hypoglycaemia on the initial blood sample after birth was associated with abnormal *short-term *outcomes (death as a consequence of severe encephalopathy and evidence of moderate to severe encephalopathy with or without seizures) in term infants with severe fetal acidaemia [9]. However no long term outcome has been reported from that group. In preterm infants, it has been shown that hypoglycaemia was associated with mental and motor development scores at age of 18 months corrected. However at age of 8 years, only arithmetic and motor scores were affected [5,25]. It has been shown that intracranial MRI abnormalities in full term infants with neonatal hypoglycaemia are resolved two months later [26]. Hypoglycaemia has been associated with abnormal neurological outcomes but not with abnormal psychomotor development [3,27,28].

Available data on the relationship between hyperglycaemia and adverse outcome is inconclusive [13,15]. In infant rats, it has been shown that hyperglycaemia during an hypoxic-ischaemic insult can have a beneficial effect against brain injury [29]. LeBlanc et al showed that, in newborn piglets, hyperglycaemia after hypoxic-ischaemic injury does not worsen the brain injury [30]. In newborn piglets, Park et al showed that brain energy metabolism was affected by hyperglycaemia during the immediate reperfusion period after hypoxic-ischaemic brain insult [21]. In fetal sheep, Blomstrand S et al showed that hyperglycemia during asphyxia reduces cerebral oxygen consumption and increases acidosis [22]. Hyperglycaemia following hypoxia-ischaemia insult was shown to be harmful, in adult rats [23].

However the glucose values studied were beyond those we see in clinical situation in humans. Despite the data on ELBW infants [13-15] and experience with older children [16,17], the ideal management of hyperglycaemia in neonatal encephalopathy remains unclear [19,20].

There are some limitations to the current study. Data was available in only 52 patients who are part of an ongoing study of continuous early EEG in HIE. The association between blood glucose and neuro-developmental outcome was not an *a priori *outcome in this study. Standard blood sampling was not possible, since in emergency situations, blood glucose samples were collected from arterial, capillary or venous blood which may independently affect glucose levels. Initial blood glucose samples were collected within 30 and 100 minutes after delivery in inborn and outborn infants respectively. These were repeated between 30 minutes and 4 hours, as clinically indicated. It is, therefore, possible that there were variations in blood glucose values that were missed in between measurements. Finally this cohort of neonates with HIE was recruited prior to introduction of therapeutic hypothermia as a standard of care. Whether variations in blood glucose will affect neuro-developmental outcome in infants treated with therapeutic hypothermia remains to be seen.

## Conclusion

We conclude that it is difficult to avoid hypoglycaemia and/or hyperglycaemia in infants with HIE, since such variations in blood glucose levels often occur soon after birth, and may be related to the asphyxial process. In term infants with HIE, glucose profiles vary widely during the first 72 hours of life. Early hypoglycaemia occurs more frequently in infants with severe HIE, and is therefore associated with abnormal outcome. The further exploration of the relationship between glycaemic control and neurological outcome will require larger numbers of patients and continuous blood glucose monitoring.

## Competing interests

The authors declare that they have no competing interests.

## Authors' contributions

MN has made contributions to collect the data from the charts and to analysis and interpretation of the data. He is the main author of the manuscript. DM participated in the design of the study, recruitment, and neurodevelopmental follow up. DM has made contributions to analysis and interpretation of the data and participated in drafted the manuscript. GB in the design of the study, infant recruitment, study and follow up. GB has participated in drafted the manuscript. ED has made contributions to analysis and interpretation of the data, manuscript draft and critical appraisal. CAR participated in the design of the study, infant recruitment and follow up. He has participated in drafting the manuscript and revising it critically for important intellectual content and has given final approval of the version to be published. All authors read and approved the final manuscript.

## Pre-publication history

The pre-publication history for this paper can be accessed here:

http://www.biomedcentral.com/1471-2431/11/10/prepub
